# Economic attractiveness of domestic rainwater harvesting in Brazilian cities

**DOI:** 10.1007/s43832-023-00033-1

**Published:** 2023-06-01

**Authors:** Marcelo Castier, Paula de Barros Barreto

**Affiliations:** 1 Chemical Engineering Program, Texas A &M University at Qatar, Education City, Doha, Qatar; 2 Facultad de Ciencias de la Ingeniería, Universidad Paraguayo Alemana, Lopez de Vega 1279, San Lorenzo, Paraguay

**Keywords:** Rainwater harvesting, Water security, Simulation, Costing, Brazil

## Abstract

Brazil is the fifth largest country by area in the world, with a variety of climates in its territory. This work assesses the economic attractiveness of domestic rainwater harvesting direct feed systems, for the replacement of non-drinking water, in 148 locations of the Brazilian territory considering hourly rainfall data for the 14-year period between 2008 and 2021. The water tariff and consumption data for each location were also considered along with estimated hourly and monthly consumption patterns. With annual operating expenses at 1% of the capital expenditure and an annual discount rate of 8%, the net present value for the 14-year period is positive in only 15 locations, which are among those with the highest water tariffs in the country. The results of these base case simulations discourage the installation of domestic RWH systems in most of the Brazilian locations considered. However, the results also show that, depending on house occupancy, filtration equipment cost, water tariff, and catchment area, outcomes more favorable to the installation of RWH systems are obtained, especially in cities of Brazil’s southern and southeastern regions.

## Highlights

- Detailed rainfall, water consumption, and tariff data for 148 Brazilian locations were used.
- Rainwater harvesting is economically attractive in a small fraction of these locations.
- Economic attractiveness of rainwater harvesting is higher in southern Brazil.

## Introduction

Domestic rainwater harvesting (RWH) systems reduce water consumption from public suppliers, especially for those uses that do not require drinking water [1]. They can also contribute to minimizing floods and torrents during heavy rain episodes, as demonstrated by studies in different parts of the world [2–6], and serve as a water reserve for when an interruption of the public water supply occurs. There are several types of incentives to install them: climatic, as in the case of remote arid and semi-arid regions [7]; legal, because of laws that impose their installation [8]; and economic, either because of their intrinsic performance or because of subsidies. In the absence of a legal obligation, the installation of a domestic RWH system depends on the decision of each individual house owner based on economic evaluations, reliability of the public water supply, perception of social and ecological benefits, or other criteria, some of which may be subjective.

The economics of a domestic RWH installation represents a complex interplay of several factors. These include the rainfall at the location, the water tariff charged by public suppliers, the size and price of the RWH tank and equipment, operating costs, and household characteristics, such as the size and type of the roof that will collect rainwater, number of occupants, and water consumption, which can vary significantly between households and even within households [9] and depend on spatial and time-dependent patterns [10, 11].

There are many examples of studies about the economics of RWH systems. A study based on public buildings of the Swinburne University of Technology campus at Hawthorn, Australia, concluded that the payback period is between 3 and 18.5 years depending on several conditions [12]. A recent study in Jordan [13] reports payback periods between 4 and 45 years, depending on the tank material and on the cost of water. A study about rainwater harvesting in a specific community in Malaysia [14] reports payback periods that range from 5.8 years under favorable conditions to nearly 60 years. Another study from Malaysia [15] calculates the payback period for the installation of an RWH system in a school building as 7.12 years. Results for RWH in dairy industries in 8 locations in Uganda [16] indicate payback periods as low as 2.12 years. Another study addressed the economics of RWH in buildings in six locations in Bangladesh [8] with payback periods between 2 and 6 years depending on topography and climate. A study in Nigeria [17] focused on optimizing storage in different types of dwellings in the city of Enugu. A study from Poland explores the possibility of using RWH for the hydroponic production of lettuces [18] and reports payback periods of about 8 years. However, also in Poland, a recent study concludes that the economic sustainability of RWH systems for domestic use is questionable [19]. Other efforts to assess the economic potential of RWH systems include the development of a shortcut method based on roof area, initial cost, and rate of return to predict the payback period [20].

As in other parts of the world, there is interest in RWH systems in Brazil [21], as demonstrated by the recent update of the national technical norm that guides their installation [22]. Brazil is the fifth largest country by area in the world and exhibits a variety of climates in its territory. Northeastern Brazil is semi-arid; the central and northern regions have equatorial and tropical climates, with frequent rainfalls; southern Brazil has a humid subtropical climate. A study about installing an RWH system in a public building in the city of Florianópolis, in the state of Santa Catarina (SC), considered several scenarios and concluded that the economic indicators were favorable in all of them [23]. Another example is the assessment of the technical and financial feasibility of installing RWH systems in two public buildings of the Federal University of Pará, in the city of Belém, with the conclusion that the payback period is in the range of 6 to 10 years [24], with most of the water being replaceable by harvested rain water. A detailed and recent study on RWH systems under different scenarios [25] used historical rainfall data of five Brazilian state capitals to rank them in terms of their failure rate, defined as the total water deficit volume divided by the total water demand volume during a selected time period. In their concluding remarks, these authors discourage the use of monthly rainfall time series and recommend the use of daily or sub-daily time series. They also discuss the duration of the time series. While it is clear that longer time series improve the statistics for RWH system design, typical constraints are the total or partial data unavailability from Brazilian sources.

It is evident from the literature that there are considerable differences in the economic attractiveness of RWH systems, which depend on climatic and economic factors and on the use of the facility, either for residential or industrial/commercial purposes. A common feature is that these published studies often focus their analyses on a single location or on a small set of adjacent locations. In this paper, the goal is to assess the economic attractiveness of domestic RWH in several locations of the Brazilian territory. In this sense, it expands recent work [23–25] by considering many more locations. To accomplish that, we use historical rainfall data on a hourly basis available for several locations in Brazil and the water tariff and consumption at each of these locations, both obtained from public databases. Because of their diversity of climate and water tariff and consumption, Brazilian cities are an interesting test ground for a systematic assessment of RWH for domestic applications.

## Methodology

In this section, we summarize information about the data and their sources and about the calculations performed in this work.

### Rainfall data

Historical rainfall data were obtained from the Brazilian National Institute of Meteorology (Instituto Nacional de Meteorologia—INMET) [26]. At the time of the writing, it contains hourly rainfall data between 2000 and 2021 for a growing number of Brazilian cities. As observed previously [25], there are gaps in the data.

To strike a balance between the number of measurement sites and the length of the data series, we used data for a period of 14 years between 2008 and 2021. In most cases, each location corresponds to one city, with exception of Rio de Janeiro, RJ, which has data for three measurement sites.

### Criteria for city selection

If, in the data set for a given city and year, the missing data amounted to less than 20% of the expected number of rainfall measurements—one every hour—the missing data were assumed to be 0 mm of rain during that hour, following a procedure adopted before [25] for the same database. Above the 20% threshold, the data set for that year was discarded and, in the economic evaluation, the result for the following year was repeated. All the locations with more than three failed yearly data sets were discarded. Only locations with an acceptable data set for the first year of the series (2008) were included. This screening process ended with a list of 148 locations (Table 2).

### Water consumption and tariff data

The average daily water consumption per person and the water tariff, in Brazilian reais, were obtained for each city in the year 2020 (the most recent data available) from the National System for Sanitation Information (Sistema Nacional de Informações sobre Saneamento) [27]. These tariffs were converted from Brazilian reais to United States dollars using the average exchange rate for 2020, which was taken as US$ 1.00 = R$ 5.156. These tariffs were considered as flat rates per cubic meter because there is no information about basic charges and consumption tiers in the database [27].

To calculate the water consumption per household, the number of occupants is necessary. The most recent official information, from the 2010 census [28], is that the average number of occupants in a Brazilian household is equal to 3.31.

Roof-harvested rain water, if untreated, may pose biological and chemical risks. For example, a study from Australia [29] shows that pathogens, likely from fecal materials of small animals with access to the roof, may be present in harvested rainwater. It suggests, for the sake of prudence, water boiling, UV or other forms of disinfection prior to use as potable water. A study from South Africa [30] mentions that the microbial quality of harvested rainwater may not always comply with drinking water standards. Further, a recent publication [31] points out that the concentration of four perfluoroalkyl acids (PFAAs)—some of the so-called forever chemicals—in rainwater exceeds the United States Environmental Protection Agency Lifetime Drinking Water Health Advisory levels. Thus, the percentage of the total domestic use of water that can be replaced by harvested rain water will depend on whether the RWH facility will treat the water to make it suitable for human consumption and personal hygiene. The assumption adopted in this work is that the treatment will not reach such levels. Therefore, it was considered that the harvested rain water will not be used for activities such as showering, hand washing, dish washing, and food preparation. It was considered that the harvested rain water can be used in toilets, clothes washing, and other applications that require little water treatment. Such uses in Brazilian households range from 19 to 40% of the domestic water consumption according to a study [32] that compiled information from three different sources. According to literature information on domestic water use in the United States of America [33], such percentage is 43%, which is the value adopted for the calculations of this work.

We also accounted for the monthly and hourly water consumption patterns. Based on literature data [34], we define $f_{M}$ and $f_{h}$ as multipliers of the average hourly water consumption, which depend on the month and on the time of the day, respectively. The monthly multiplier $f_{M}$ embeds a six-month shift with respect to the original data, which is based on the northern hemisphere, because most Brazilian cities are in the southern hemisphere. Table 1 shows the values adopted.

**Table 1:** Multipliers for the hourly ($f_h$) and monthly ($f_M$) water consumption patterns

Hour	$f_h$	Hour	$f_h$	Month	$f_M$
0	0.141	12	0.692	1	1.435
1	0.102	13	0.640	2	1.233
2	0.090	14	0.640	3	1.156
3	0.102	15	0.666	4	0.758
4	0.231	16	2.510	5	0.752
5	2.254	17	2.510	6	0.758
6	2.536	18	2.510	7	0.783
7	2.651	19	0.653	8	0.706
8	0.922	20	0.615	9	0.758
9	0.909	21	0.551	10	1.109
10	0.768	22	0.359	11	1.224
11	0.743	23	0.205	12	1.326

### Capital and operating expenses

The capital expenditure, *I*, in equipment was assumed to be equal to the summation of the costs of three units: a pump, a filter kit for RWH systems, and a polyethylene storage tank. These costs were obtained in Brazilian reais from local vendors and converted to US dollars using the exchange rate of the day. The cost of installing the RWH system was neglected.

The prices of a 1/2 HP pump [35] and of the filter kit [36] are US$ 127.78 and US$ 390.35, respectively. The prices of [500, 1000, 2000, 5000, 10,000, 20,000] L polyethylene tanks are US$ [69.10, 112.22, 272.83, 483.59, 938.34, 2515.47], respectively [37].

The value of the annual operating expenses, *OPEX*, is calculated as a fraction $f_e$ of the capital expenditure:

1 $$\begin{aligned} OPEX = f_e I \end{aligned}$$

### Contingencies

In addition to its regular role as part of the RWH system, it is assumed that the tank can have the additional role of storing water for emergency uses when there is an interruption of the public water supply. To consider that, we specify $V_{min}$ as the minimum volume of water allowed in the tank during its normal operation.

### Water balance in the tank

There are two main types of RWH systems: gravity feed systems, in which the harvested water is pumped to a header tank from where it flows by gravity when needed, and direct feed systems, in which the harvested water is pumped directly to the point of consumption. A review paper on energy intensity in RWH systems, with authors from Australia and Brazil [38], concludes that gravity feed systems have the potential to be more energy-efficient than direct feed systems. Other sources [39, 40] present a different view and point out that, for maximum reuse of rainwater, direct feed systems tend to be more efficient. A recent study from Brazil [41] claims that direct feed systems are more energy-efficient but concluded that gravity and direct feed systems led to similar paybacks for RWH systems in the city of Maringá, in the State of Paraná. Here, it is assumed that direct feed systems will be used.

As we consider that the water density is constant, the mass balance of water in the tank is replaced by a volumetric balance. The initial volume of water inside the tank is $V_0$. At the end of any subsequent time interval *i*, the volume of water in the tank, $V_i$, is:

2 $$\begin{aligned} V_i=V_{i-1}+\Delta V_i \end{aligned}$$

The symbol $\Delta V_i$ represents the change in volume of water in the tank during interval *i*.

3 $$\begin{aligned} \Delta V_i= (\Delta V_i)_{in} - (\Delta V_i)_{out} \end{aligned}$$

with:

4 $$\begin{aligned} (\Delta V_i)_{in}= & {} H_i A D \end{aligned}$$

5 $$\begin{aligned} (\Delta V_i)_{out}= & {} f_{M,i} f_{h,i} {\dot{w} _{h}} \Delta t_i \end{aligned}$$

In these equations, $H_i$ represents the rainfall in during interval *i*, *A* is the roof catchment area, and *D* is its drainage coefficient [42], which is the fraction of the rainwater collected on the roof. The symbol $\dot{w}_{h}$ represents the average non-drinking water consumption per household per hour and $\Delta t_i$ is the time interval in the rainfall measurements, which is of one hour in this work.

If $V_i < V_{min}$, $V_i$ is set to $V_{min}$, with the water deficit covered by the public supplier. If $V_i$ is larger than the tank’s capacity, $V_{max}$, $V_i$ is set to $V_{max}$, and at least part of the rainfall during interval *i* is not harvested. In this case, the volume harvested during interval *i* is:

6 $$\begin{aligned} (\Delta V_i)_{in}= V_{max} - V_{i-1} + (\Delta V_i)_{out} \end{aligned}$$

Therefore, $(\Delta V_i)_{in}$, calculated either with Eq. 4 or 6, is the volume of water harvested during interval *i*.

### Performance indicators

To analyze the results, we use the harvest/demand ratio of non-drinking water and net present value of the RWH system. The calculations are based on historical rainfall data during a period of 14 years, between 2008 and 2021, to project the performance an RWH system would have during its operation. We neglect the effect of inflation and assume the investment is done in year 0, that is, in 2007.

#### Harvest/demand ratio of non-drinking water

The harvest/demand ratio of non-drinking water ($R_{HD}$) is calculated dividing the volume of water harvested during a period of $n_p$ time intervals by the non-drinking water consumption during the same period:

7 $$\begin{aligned} {R_{HD}} = \frac{{\sum \nolimits _{i = 1}^{{n_p}} {{{\left( {\Delta {V_i}} \right) }_{in}}} }}{{\sum \nolimits _{i = 1}^{{n_p}} {{{\left( {\Delta {V_i}} \right) }_{out}}} }} \end{aligned}$$

It is possible that $R_{HD}>1$ in a given period and this indicates that more water is harvested than utilized during the period. This water surplus stays in the tank for later use.

#### Net present value

The net present value (*NPV*) is calculated using:

8 $$\begin{aligned} NPV = - I + \sum \limits _{j = 1}^{{n_y}} {\frac{{{S_j}}}{{{{\left( {1 + r} \right) }^j}}}} \end{aligned}$$

where $n_y$ is the number of years, *r* is the annual discount rate, and $S_j$ is the savings in year *j*, which is the difference between the savings in water bills and the operating expenses (Eq. 1):

9 $$\begin{aligned} {S_j} = C\sum \limits _{i = 1}^{{n_{p,j}}} {{{\left( {\Delta {V_i}} \right) }_{in}}} - OPEX \end{aligned}$$

where $n_{p,j}$ is number of time intervals in year *j* and *C* is the water tariff in a given location. We use the symbol $NPV_{14}$ to represent the net present value for the 14-year period considered in this work.

### Computational implementation

All the calculations described in this section were carried out using a program in Python, developed in-house, that takes advantage of parallelism to speed up its execution.

## Results

This section is divided into subsections, the first of which presents base cases that disregard operating costs and discount rates. These are considered in subsequent subsections. Then, there are subsections about the effects of occupancy, filtration equipment cost, water tariffs, and catchment area.

### Base cases

As of 2019, 85.6% of the permanent private households in Brazil were houses [43], with tile roofs used in 81.9% of them [44]. Based on these data, the model home adopted in this work is a house with tile roof. The average drainage coefficient calculated using the values and ranges reported in reference [45] is equal to 0.84. This value is in very good agreement with the value adopted in our calculations $-$ 0.85—which is the mid-value of the interval [0.8, 0.9] for tile roofs according to Biswas and Mandal [46]. Using values smaller than 0.85 for the drainage coefficient would lead to a more conservative analysis because of the smaller fraction of the rainwater collected on the roof; using values larger than 0.85 would have the opposite effect.

The average area of the 10% most expensive houses financed in Brazil was equal to 136 m^2^ in 2021 [47]. This upper market tier probably has the most discretionary income to invest in the installation of an RWH system. To that area, we added 10% to account for the roof’s eaves, obtaining a total catchment area of 150 m^2^, which is the value used in our base cases.

**Fig. 1:**
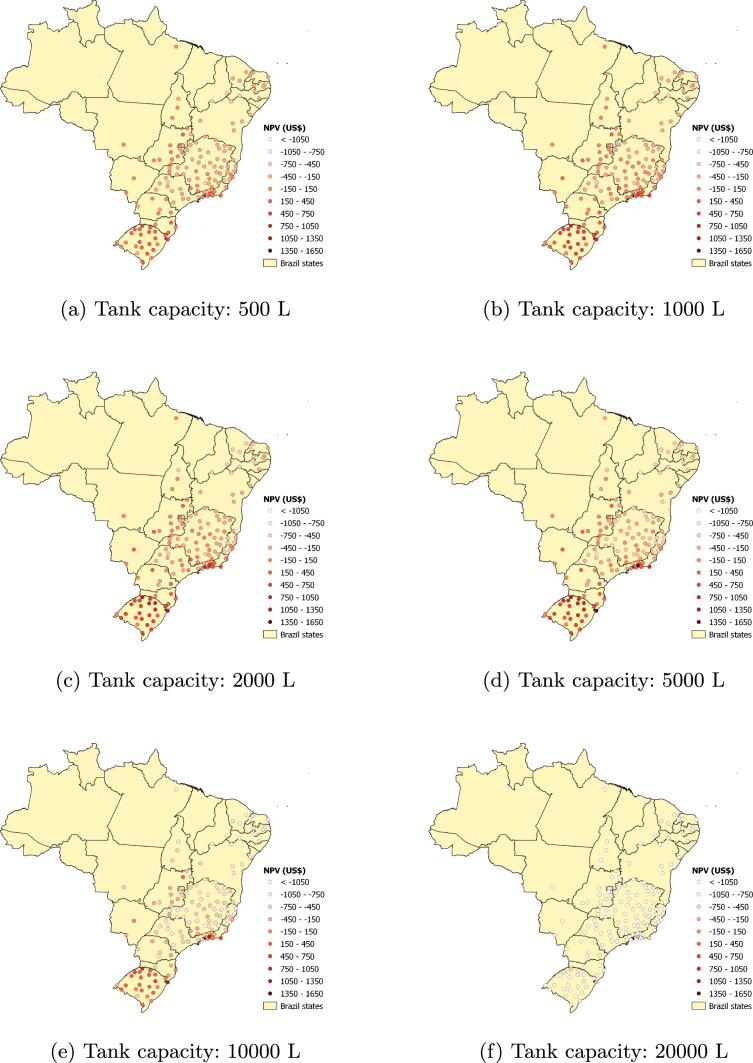
Effect of tank capacity on the net present value for the 14-year period. Catchment area: 150 m^2^; drainage coefficient: 0.85; number of occupants: 3.31 occupants; minimum volume allowed: 10% of the tank capacity; operating expenses: US$ 0.00; annual discount rate: 0%

A household with 3.31 occupants is assumed. This is the average number of occupants according to Brazil’s most recent census. The minimum and initial volumes of water in the tank are 10% and 50% of the tank’s capacity, respectively. The operating expenses and the discount rate are both equal to zero. Figure 1, plotted using maps from the Brazilian Institute of Geography and Statistics [48] loaded into the QGIS geographic information system software version 3.26.2 [49], displays the $NPV_{14}$. Points with lighter colors have small $NPV_{14}$ (negative in several cases) and those with more intense colors have large $NPV_{14}$. Overall, there is a substantial scattering of the $NPV_{14}$, depending on the location, which indicates a large disparity in the economic attractiveness of installing a domestic RWH system.

In Fig. 1, there are very few points in the northern part of the country, which mostly corresponds to the Amazon region. This results from the few rainfall data sets for this part of the country that passed our completeness tests for inclusion in this study. Most of the locations that passed these tests are in the northeastern, southeastern, southern regions, which have the largest population densities.

Another observable trend in Fig. 1 is that, for all tank capacities, the $NPV_{14}$ is small, and often negative, in the northeastern cities, despite the fact that some of them are in a semi-arid region. This is a consequence of low water consumption and low water tariffs. On the other hand, the southernmost state, Rio Grande do Sul (RS), has some of the largest $NPV_{14}$ because of the high water tariff in many of its cities, possibly because this affluent state occasionally undergoes periods of severe drought.

The number of times the [500, 1000, 2000, 5000, 10,000, 20,000] L tanks lead to the largest $NPV_{14}$ is respectively equal to [7, 99, 16, 26, 0, 0]. Therefore, a 1000 L tank would be the most favorable solution in 66.9% of the 148 locations considered. The largest tanks, of 10,000 L and 20,000 L, are too costly for the conditions of this case study and never provided the best solution for any given location.

There are 58 locations where at least one tank capacity leads to a positive $NPV_{14}$. Among them, the 1000 L tank gives the best solution 19 times, while the numbers of times for the 2000 L and 5000 L tanks are 14 and 25, respectively. Interestingly, among the 25 locations with the largest $NPV_{14}$, the best solutions are achieved 6 times with the 2000 L tank and 19 times with the 5000 L tank. The general observation is that, for conditions that favor a positive $NPV_{14}$, the best tank capacities tend to be either 2000 L or 5000 L, depending on the specific conditions of the location.

**Fig. 2:**
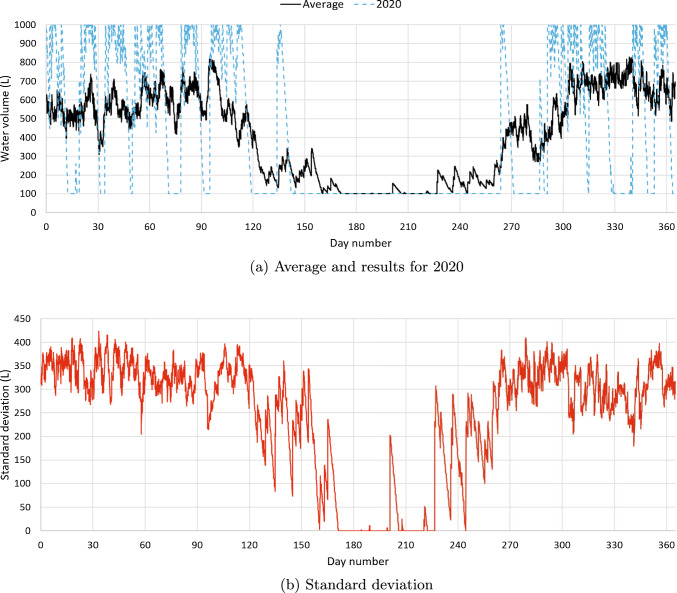
Volume water inside a 1000 L tank in Brasília as a function of the day of the year: average values, values in 2020, and standard deviations. Catchment area: 150 m^2^; drainage coefficient: 0.85; number of occupants: 3.31 occupants; minimum volume allowed: 10% of the tank capacity

To illustrate the results of our simulations, Fig. 2 shows the volume of water inside a 1000 L tank as a function of the day of the year in Brasília, which is Brazil’s capital city and selected because its rainfall data set is quite complete. Based on the $NPV_{14}$, the 1000 L tank is the best option in Brasília. In Fig. 2a, the solid line represents the average volume of water in the tank, which was calculated as follows: for each date and time, the hourly values for each of the 14 years were averaged. The values for 29 February in the leap years were discarded. The curve for the average is deceitful in the sense that it might give the impression that the tank never fills up during the year, but this is not correct. Figure 2a includes the results for 2020, which was chosen arbitrarily. Despite the effects of the COVID-19 pandemic during 2020, official data [50] show that the per capita water consumption for that year in the Brasília region was within 0.2% of the average for the period 2017–2020. The results for 2020 show that the tank fills up and empties to the minimum acceptable volume of 100 L several times during the year. Figure 2b shows the standard deviations, which are quite large, with exception of a period of about 50 days in the middle of the year, when Brasília is notoriously dry. During this period, it is almost certain that the water in the tank will be at its minimum acceptable level.

**Fig. 3:**
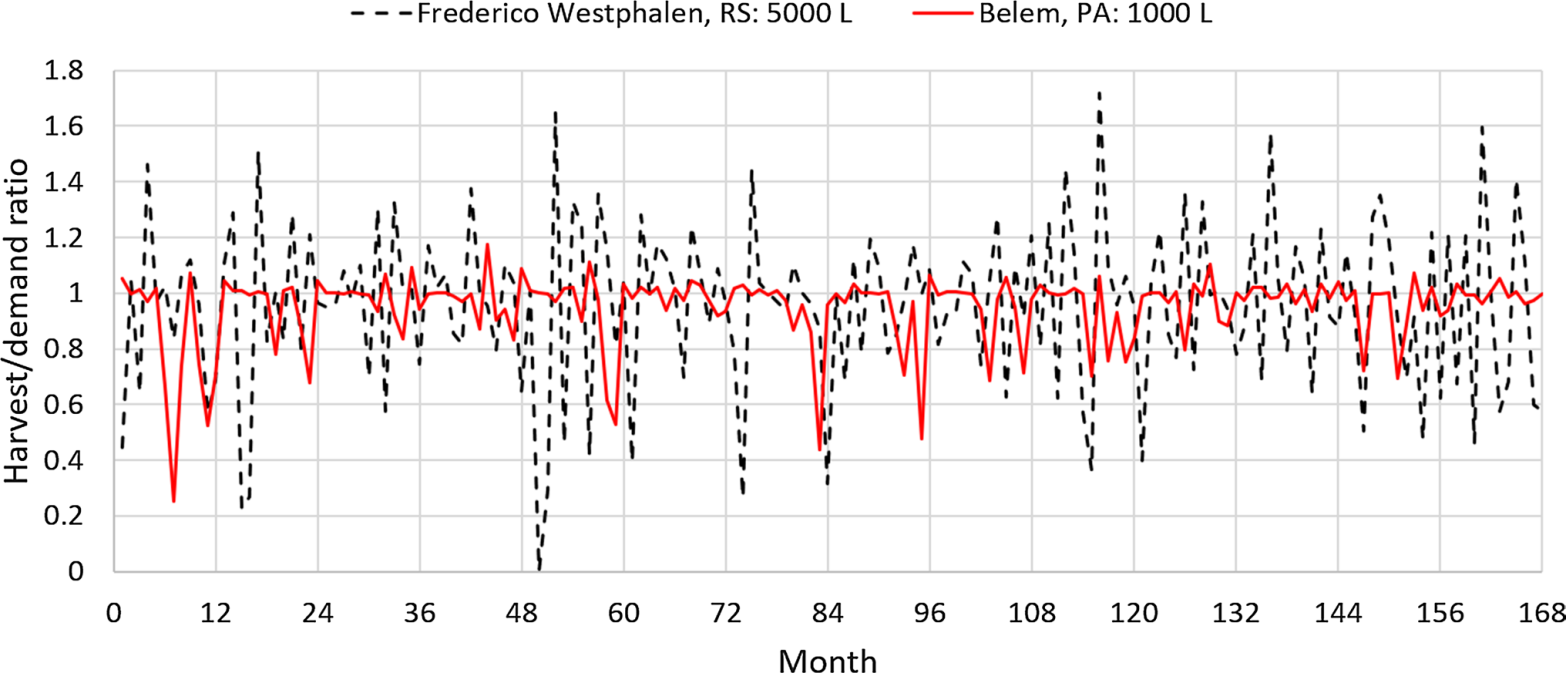
Harvest/demand ratio of non-drinking water. Catchment area: 150 m^2^; drainage coefficient: 0.85; number of occupants: 3.31 occupants; minimum volume allowed: 10% of the tank capacity. Dashed line: Frederico Westphalen, RS; solid line: Belém, PA

**Fig. 4:**
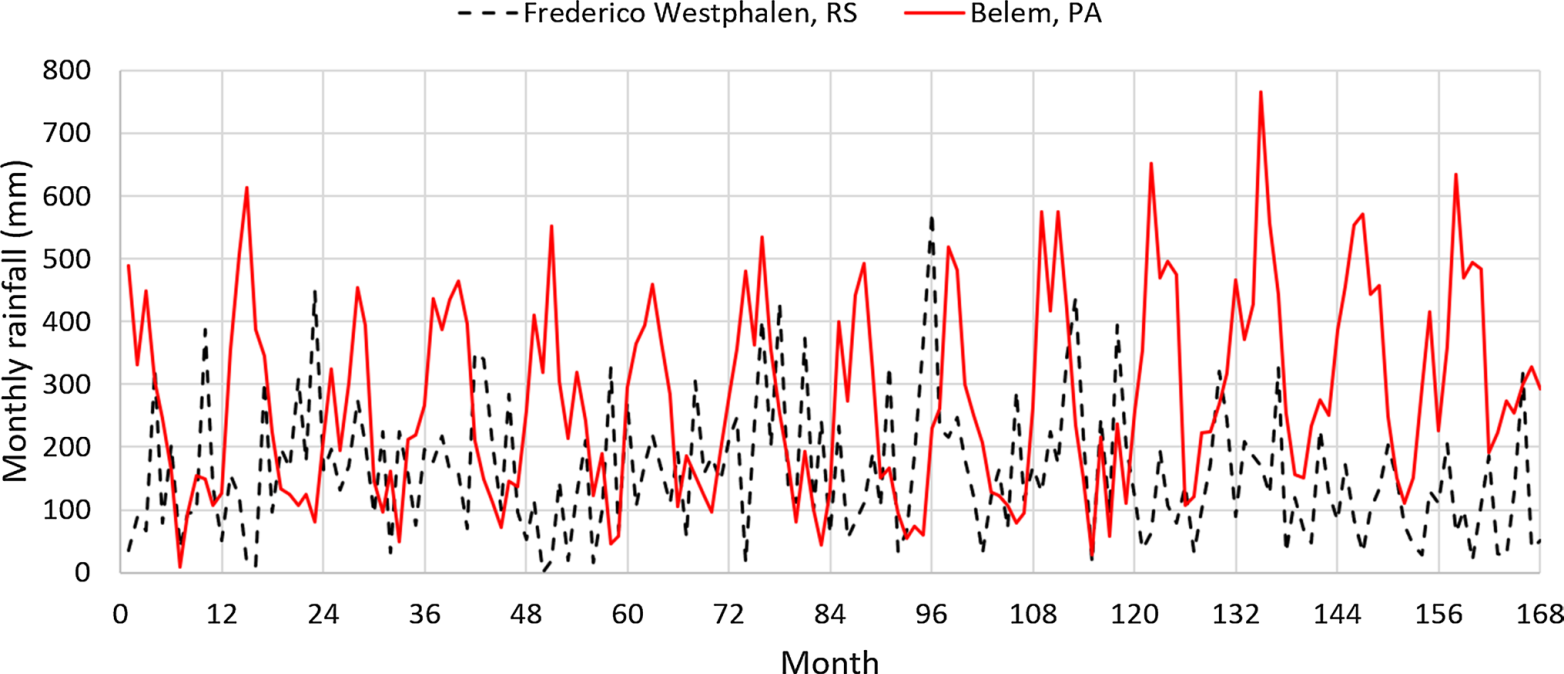
Average monthly rainfall. Solid line: Belém, PA. Dashed line: Frederico Westphalen, RS

Next, results for the cities of Belém, in the State of Pará (PA), and of Frederico Westphalen, in the State of Rio Grande do Sul (RS), are compared. The choice of these two cities, which are about 3500 km apart, is arbitrary but they have several contrasting factors. Belém is in the northern region and is the rainiest state capital in Brazil. Frederico Westphalen is located in the southernmost, drought-prone Brazilian state, where water tariffs are high. Unlike Belém, Frederico Westphalen ranked among the most favorable locations for the installation of an RWH system according to our calculations. Fig. 3 compares the harvest/demand ratio of non-drinking water for the case of a city with a tank of 1000 L (Belém, in the State of Pará (PA)) and another with a tank of 5000 L (Frederico Westphalen, in the State of Rio Grande do Sul (RS)). These capacities are the most favorable for these cities based on the $NPV_{14}$. The average $R_{HD}$ for the whole period is 0.947 in Belém and 0.954 in Frederico Westphalen, that is, about 95% of the non-drinking water demand is fulfilled by the harvested rainwater. Figure 3 also shows that the fluctuation in $R_{HD}$ is larger in Frederico Westphalen than in Belém. In the former, because of the larger tank capacity, the harvested volume may be much more than the demand in rainy months and, in dry months, the harvested volume may be much less than the demand. To analyze this difference, it is useful to observe the average monthly rainfalls, which are compared in Fig. 4. During the period considered, the average annual rainfall was 3334 mm in Belém and 1906 mm in Frederico Westphalen. With a smaller water demand per occupant and plenty of rain, the best tank capacity for an RWH system in Belém is 1000 L, which supplies 95% of the non-drinking water needs. With less rain and a higher water demand per occupant, an RWH system in Frederico Westphalen needs a larger tank to fulfill the same percentage of the local demand.

**Fig. 5:**
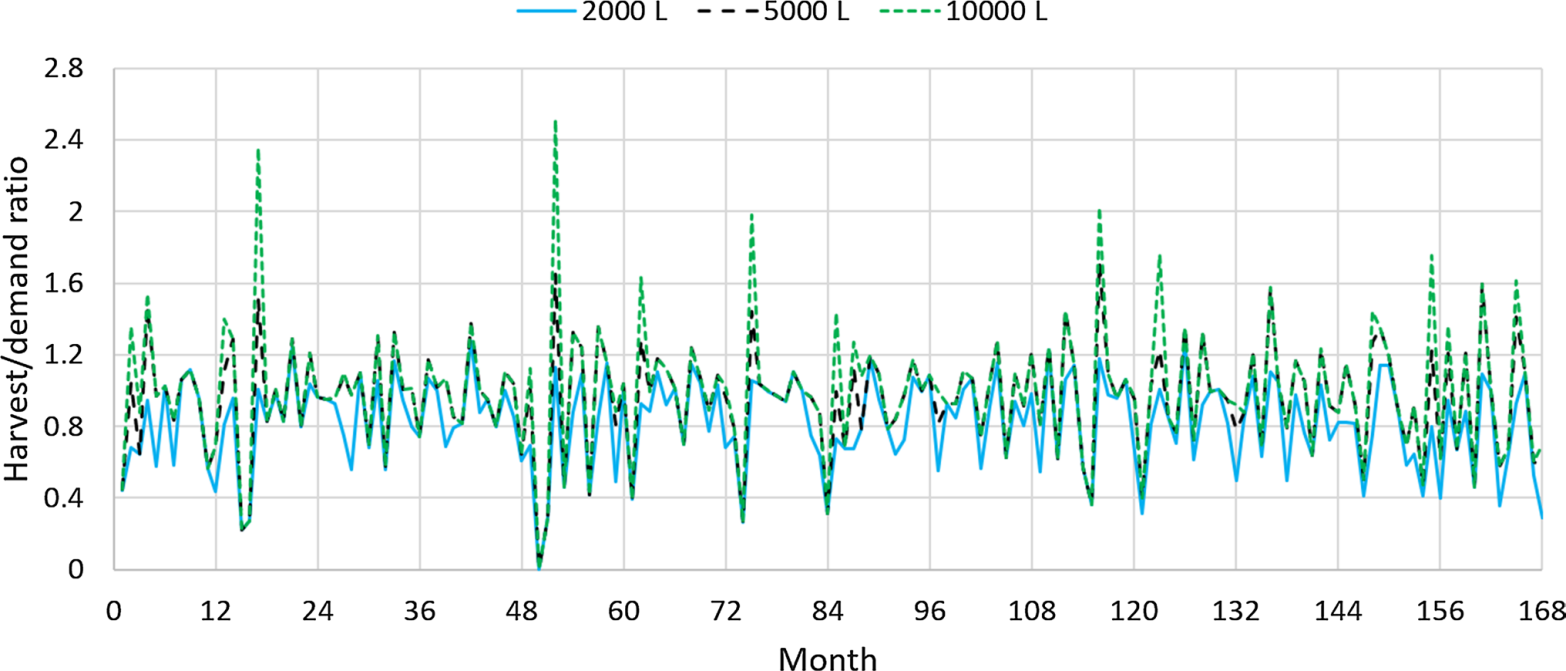
Effect of tank capacity on the harvest/demand ratio of non-drinking water in the city of Frederico Westphalen, RS. Catchment area: 150 m^2^; drainage coefficient: 0.85; number of occupants: 3.31 occupants; minimum volume allowed: 10% of the tank capacity. Solid line: 2000 L; dashed line: 5000 L; dotted line: 10,000 L

Figure 5 shows the the harvest/demand ratio of non-drinking water in the city of Frederico Westphalen for the tank capacities of 2000 L, 5000 L, and 10,000 L, whose average $R_{HD}$ for the whole period are 0.819, 0.954, and 0.996, respectively. There are periods when the curves for the 5000 L and 10,000 L tanks overlap—and even some periods when all three curves overlap—during which the tank capacity is not the limiting factor for RWH. As the tank capacity increases, the peaks in the harvest/demand ratio become more pronounced because more water can be stored during rainy periods. The largest average harvest/demand ratio, $R_{HD}=0.996$, is for 10,000 L tank but its $NPV_{14}$ is less than that for the 5000 L tank, whose average harvest/demand ratio is $R_{HD}=0.954$.

### Accounting for operating expenses

**Fig. 6:**
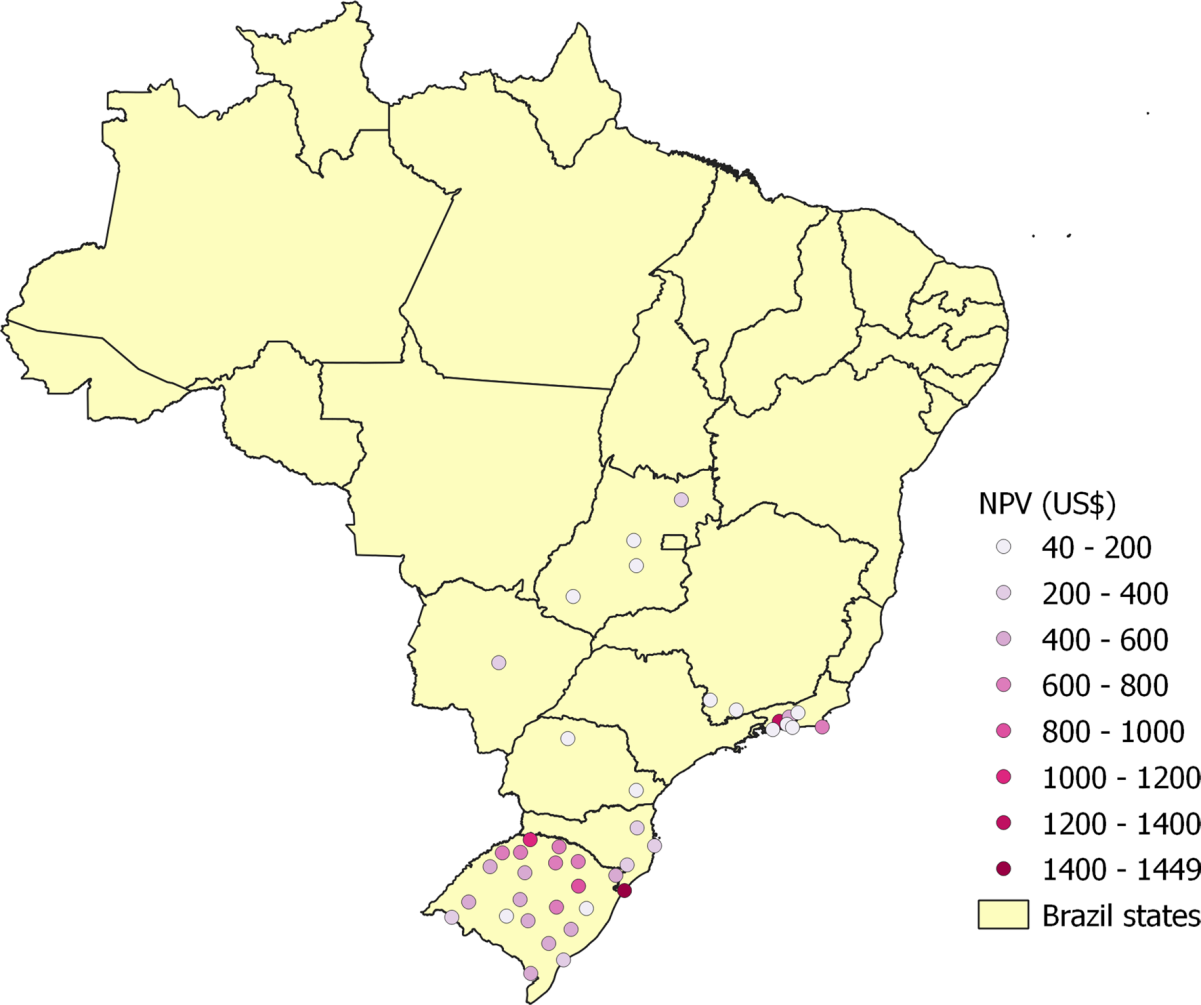
Locations with positive net present value for the 14-year period when accounting for annual operating expenses equal to 1% of the capital expenditure. Catchment area: 150 m^2^; drainage coefficient: 0.85; number of occupants: 3.31; minimum volume allowed: 10% of the tank capacity

Next, we consider the effect of the operating expenses. As this decreases the $NPV_{14}$, this calculation was done only for the 58 locations that had positive $NPV_{14}$ in the previous subsection. It is assumed that the annual operating expenses are 1% of the capital expenditure. Figure 6 shows the results, with the 41 locations where RWH systems would have positive $NPV_{14}$. Remarkably, no city of the northern and northeastern regions appears and most cities are in the southernmost state of Rio Grande do Sul. A tank with capacity of 1000 L is the best option in 9 of these locations, 2000 L is the best tank in 11 locations, and 5000 L is the best option in 21 locations.

### Accounting for discount rates

For the 41 locations with positive $NPV_{14}$ when the operating expenses are 1% of the capital expenditure, we also consider the effect of the discount rate for tanks with capacities of 1000 L, 2000 L, and 5000 L.

**Fig. 7:**
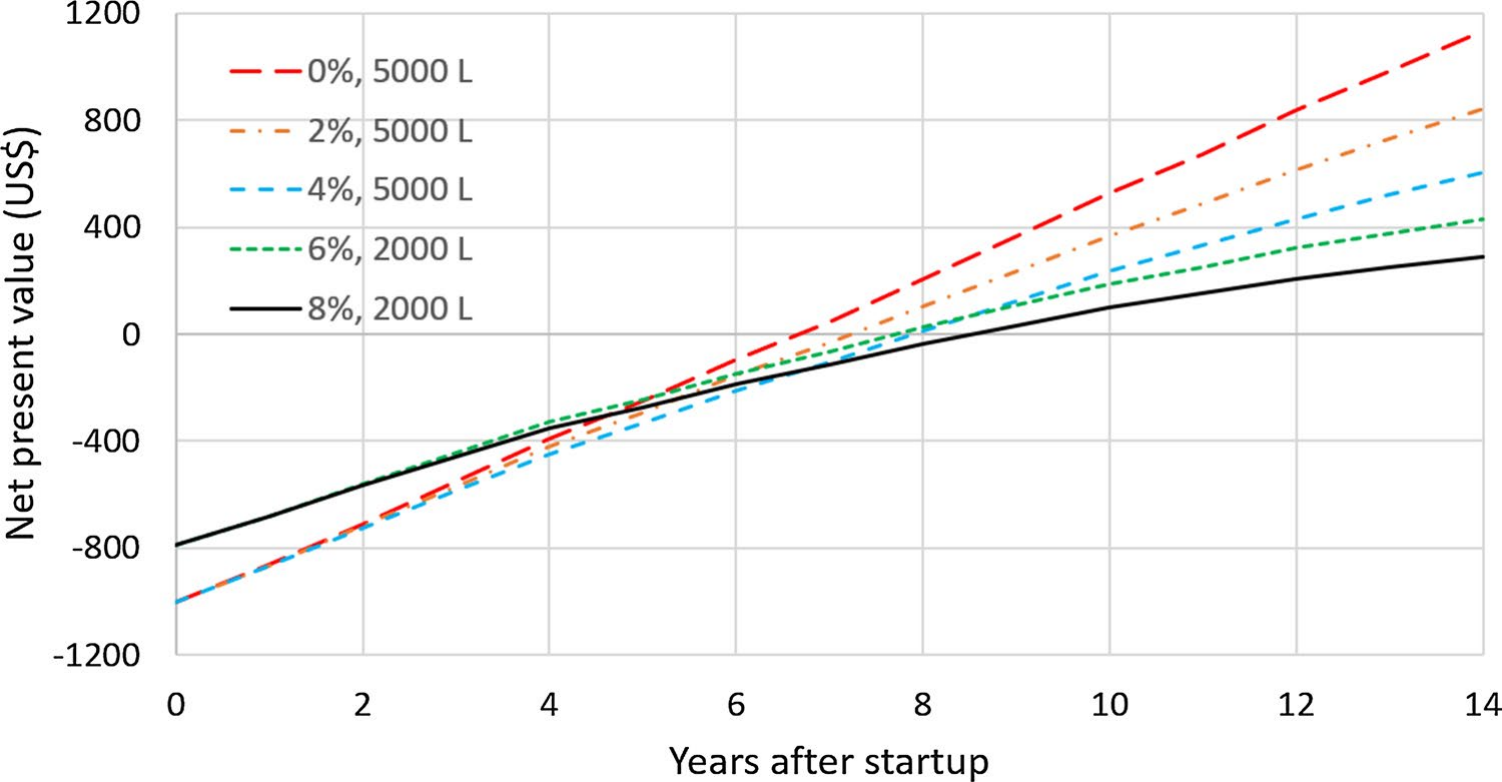
Net present value in the city of Frederico Westphalen, RS, for the several discount rates. Annual operating expenses: 1% of the capital expenditure. Catchment area: 150 m^2^; drainage coefficient: 0.85; number of occupants: 3.31; minimum volume allowed: 10% of the tank capacity

**Fig. 8:**
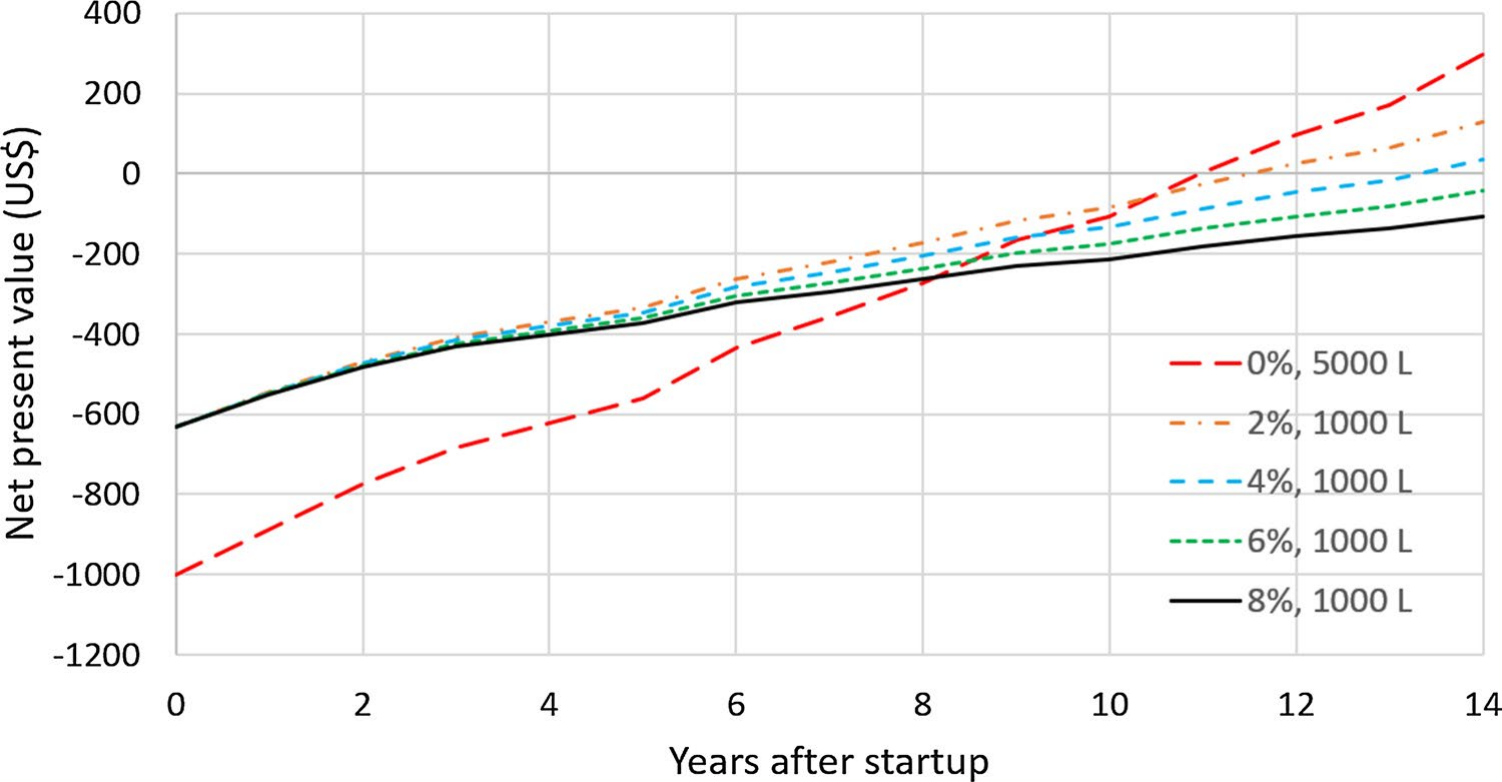
Net present value in the city of Alto Paraíso de Goiás, GO, for the several discount rates. Annual operating expenses: 1% of the capital expenditure. Catchment area: 150 m^2^; drainage coefficient: 0.85; number of occupants: 3.31; minimum volume allowed: 10% of the tank capacity

**Fig. 9:**
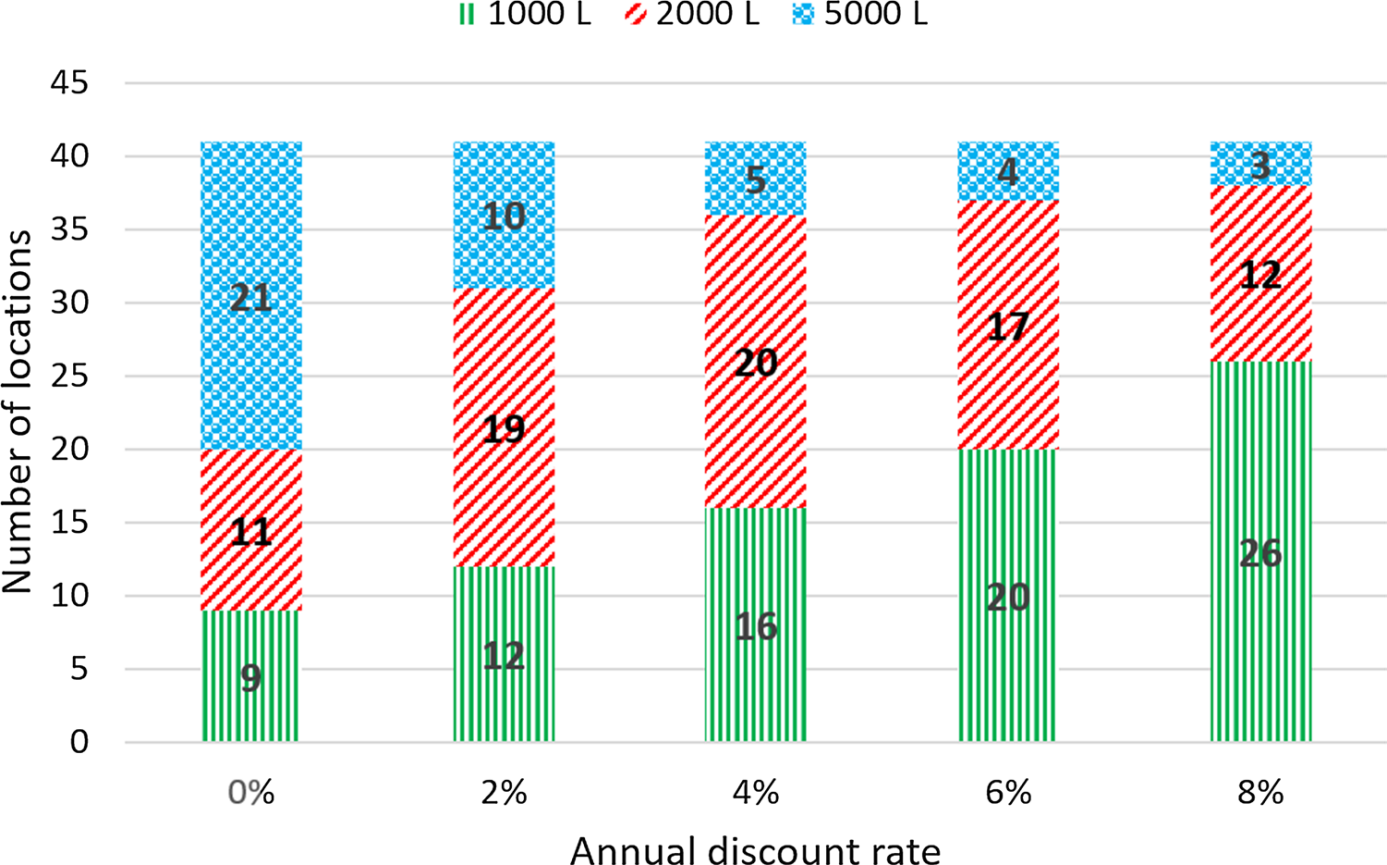
Effect of the annual discount rate on the best tank capacity. Annual operating expenses: 1% of the capital expenditure. Catchment area: 150 m^2^; drainage coefficient: 0.85; number of occupants: 3.31; minimum volume allowed: 10% of the tank capacity

**Fig. 10:**
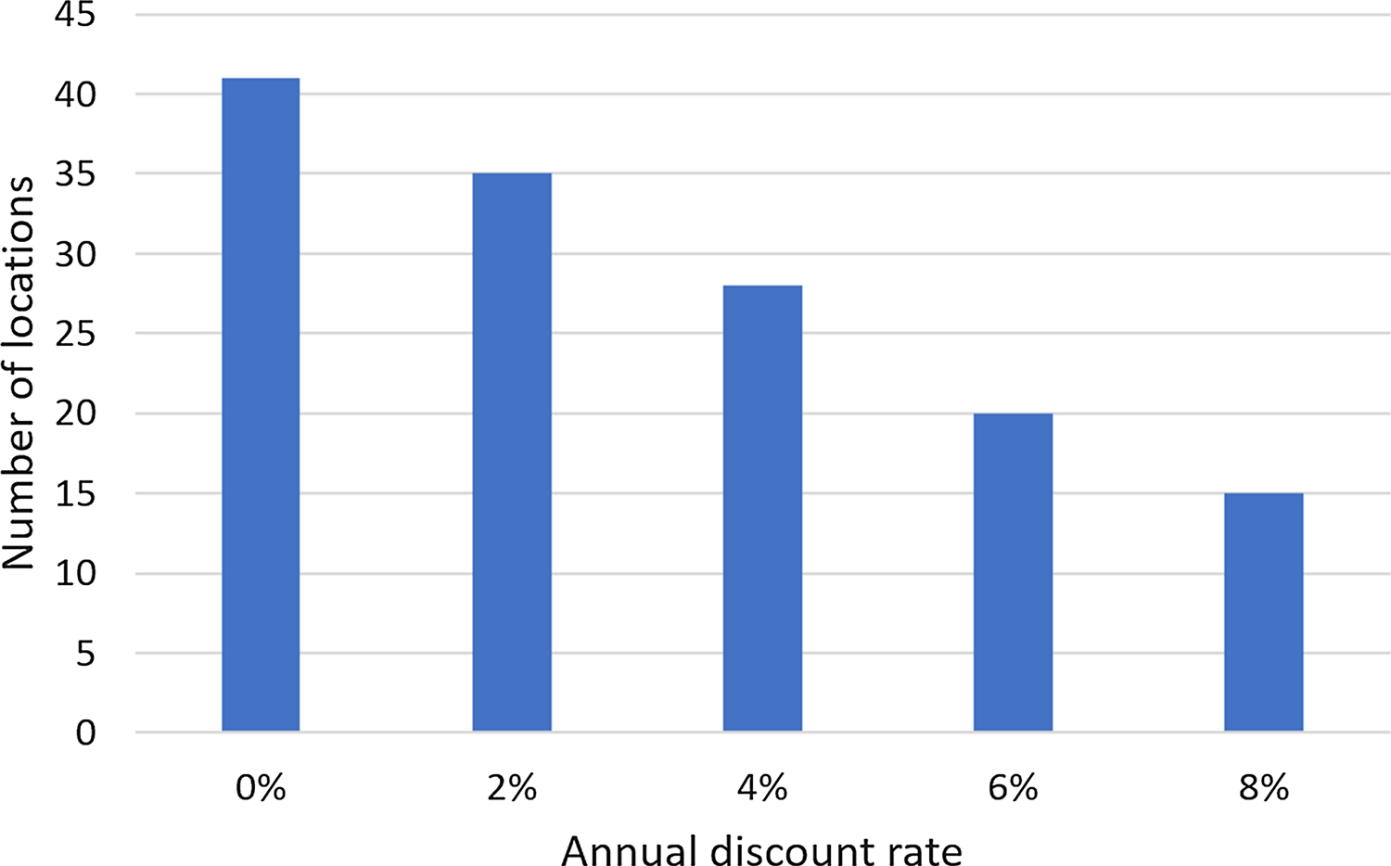
Effect of the annual discount rate on the number of locations with positive net present value for the 14-year period. Annual operating expenses: 1% of the capital expenditure. Catchment area: 150 m^2^; drainage coefficient: 0.85; number of occupants: 3.31; minimum volume allowed: 10% of the tank capacity

As an example, Fig. 7 shows the net present value in the city of Frederico Westphalen as a function of the number of years after the startup of the RWH system for different values of the discount rate. In all cases, the NPV for 14 years of operation is positive and the payback period is in the interval [6, 9] years, depending on the discount rate. The best tank capacity also depends on the discount rate. For the discount rates of 0%, 2%, and 4%, the best tank capacity is 5000 L and the project value at startup is equal to (–US$ 1001.72). For the discount rates of 6% and 8%, the 2000 L is better and the project value at startup is equal to (–US$ 790.96). Figure 8 displays analogous results for the city of Alto Paraíso de Goiás, GO. With 0% discount rate, the best tank capacity is 5000 L but, for the other discount rates, the 1000 L gives the largest $$NPV_{14}$$. For the discount rates of 0%, 2%, and 4%, the $$NPV_{14}$$ is positive but it is negative for the 6% and 8% discount rates. For both locations, increasing the discount rate favors the downsizing of the RWH system. In fact, this is a general trend, as shown in Fig. 9. It displays the number of cases in which each tank capacity—1000 L, 2000 L, or 5000 L—gives the best $$NPV_{14}$$ for the 41 locations considered. As the annual discount rate increases, the number of locations where the 5000 L tank is the best option decreases; the number of locations where the 1000 L tank is the best option increases; for the tank with the intermediate capacity of 2000 L, this number increases and then decreases. Another effect of increasing the annual discount rate is the decrease in the number of locations that have a positive $$NPV_{14}$$, as shown in Fig. 10. With a discount rate of 8%, RWH systems in only 15 of the initial 148 locations have a positive $$NPV_{14}$$. Out of these 15 locations, 13 are in the State of Rio Grande do Sul and 2 are in the State of Rio de Janeiro. Their most striking common feature is that they are among the top 22 locations with the highest water tariffs.

Based on these results for this sample of 148 locations, a 150 m^2^ roof area, the Brazilian average of 3.31 occupants, and additional assumptions of these base case simulations, a house owner would typically have economic incentive to install an RWH system in few Brazilian cities. Under such scenario, the decision to install an RWH system would result from considering additional factors, such as water security and the perception of contributing to community efforts to reduce the impact of floods and water torrents. The cities with the most favorable conditions for domestic RWH are generally located in the southernmost State of Rio Grande do Sul, which is an interesting outcome because this state is prone to suffer periods of drought. Despite the use of different indicators, this result agrees with literature findings [25] that point at Porto Alegre, the state’s capital city, as having the greatest potential for RWH among four other Brazilian state capitals. It is also appropriate to comment that the economic attractiveness of domestic RHW systems has been questioned in other parts of the world, as in Eastern Poland [19].

### Effect of occupancy

**Fig. 11:**
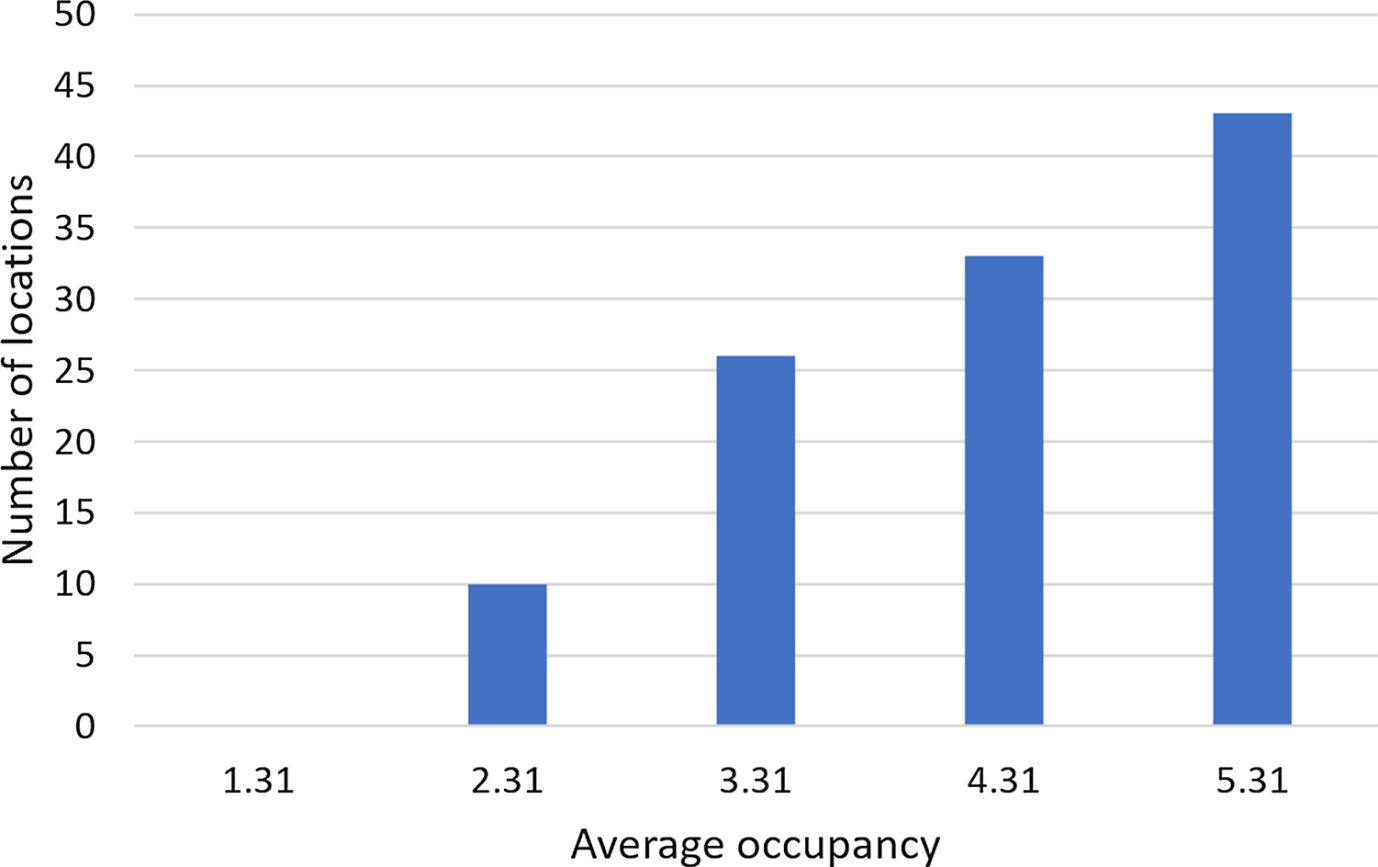
Effect of occupancy on the number of locations with positive net present value for the 14-year period. Annual operating expenses: 1% of the capital expenditure. Catchment area: 150 m^2^; drainage coefficient: 0.85; number of occupants: 3.31; tank capacity: 2000 L; minimum volume allowed: 10% of the tank capacity; annual discount rate: 4%

The domestic water consumption is calculated in this work multiplying the number of occupants by the location-dependent per capita consumption [27], and then assuming that a fraction (43%) of this consumption can be replaced by harvest rainwater. Therefore, studying the effect of occupancy is tantamount to studying the effect of water consumption. We simulated cases with $\pm \,1$ and $\pm \,2$ occupants from the national average value of 3.31. With the specifications given in its caption, Fig. 11 shows the effect of the average occupancy on the number of locations with positive $NPV_{14}$. With more occupants, the domestic water consumption grows, increasing the number of locations that benefit from the installation of RWH systems. This observation is aligned with literature results [51] indicating that the number of occupants is correlated with tendency to adopt domestic RWH systems.

### Effect of the filtration equipment cost

**Fig. 12:**
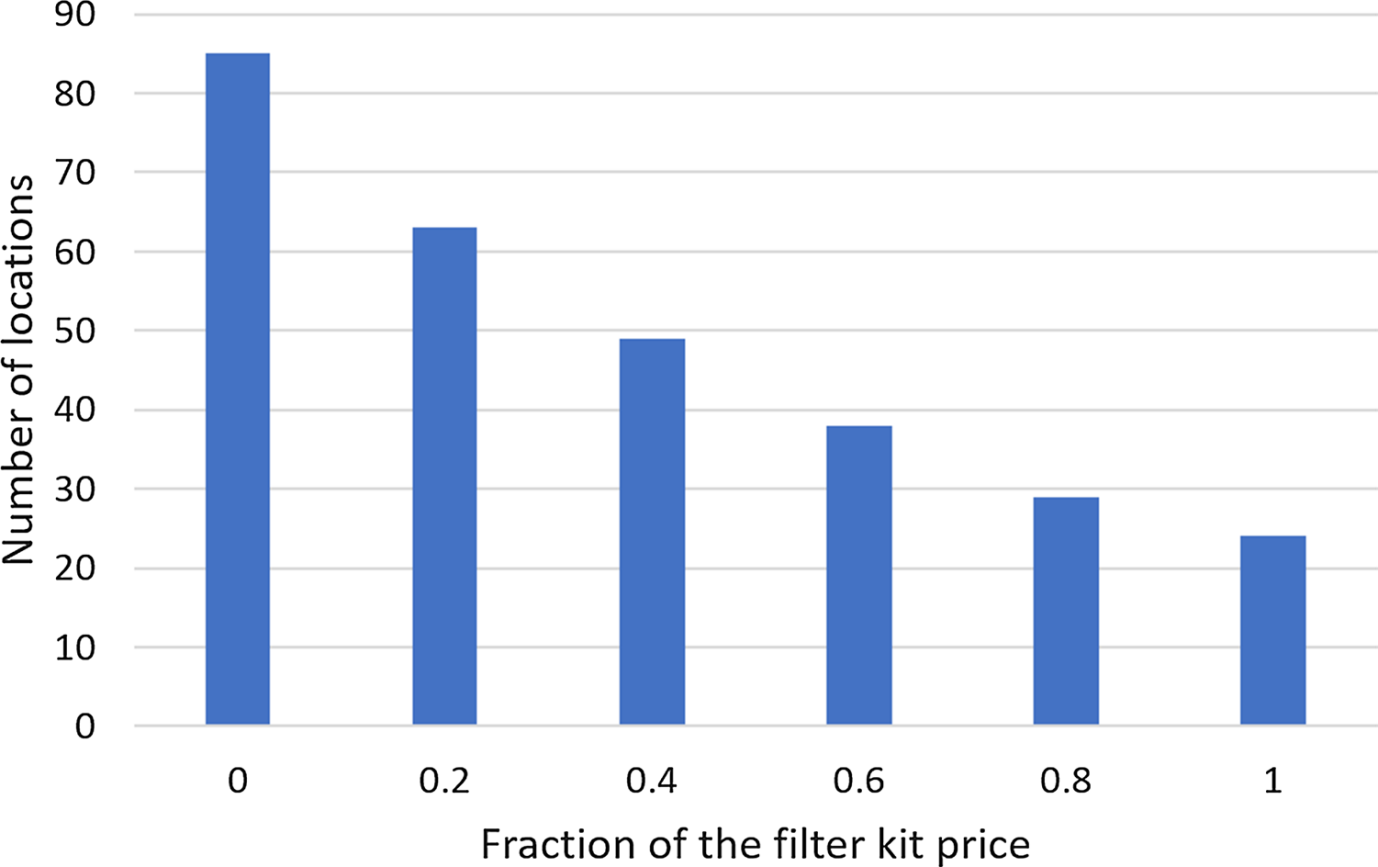
Effect of the cost fraction of the filter kit on the number of locations with positive net present value for the 14-year period. Annual operating expenses: 1% of the capital expenditure. Catchment area: 150 m^2^; drainage coefficient: 0.85; number of occupants: 3.31; tank capacity: 2000 L; minimum volume allowed: 10% of the tank capacity; annual discount rate: 4%

The base case calculations were done using the cost of off-the-shelf equipment, readily available from major suppliers in the Brazilian market. It might be possible to reduce the cost of equipment by using filtration systems developed onsite at a fraction of the kit price. For the set of specifications informed in its caption, Fig. 12 shows the effect of the filtration equipment cost on the number of locations with positive $NPV_{14}$, including the limiting case of a zero-cost filtration system. For the original filter kit price, 16.2% of the locations considered in this work have positive $NPV_{14}$; for a price fraction of 0.2, this percentage increases to 42.6%, with locations in 11 states, mostly in the southern and southeastern regions, and the Federal District (Brasília region).

### Effect of water tariffs

**Fig. 13:**
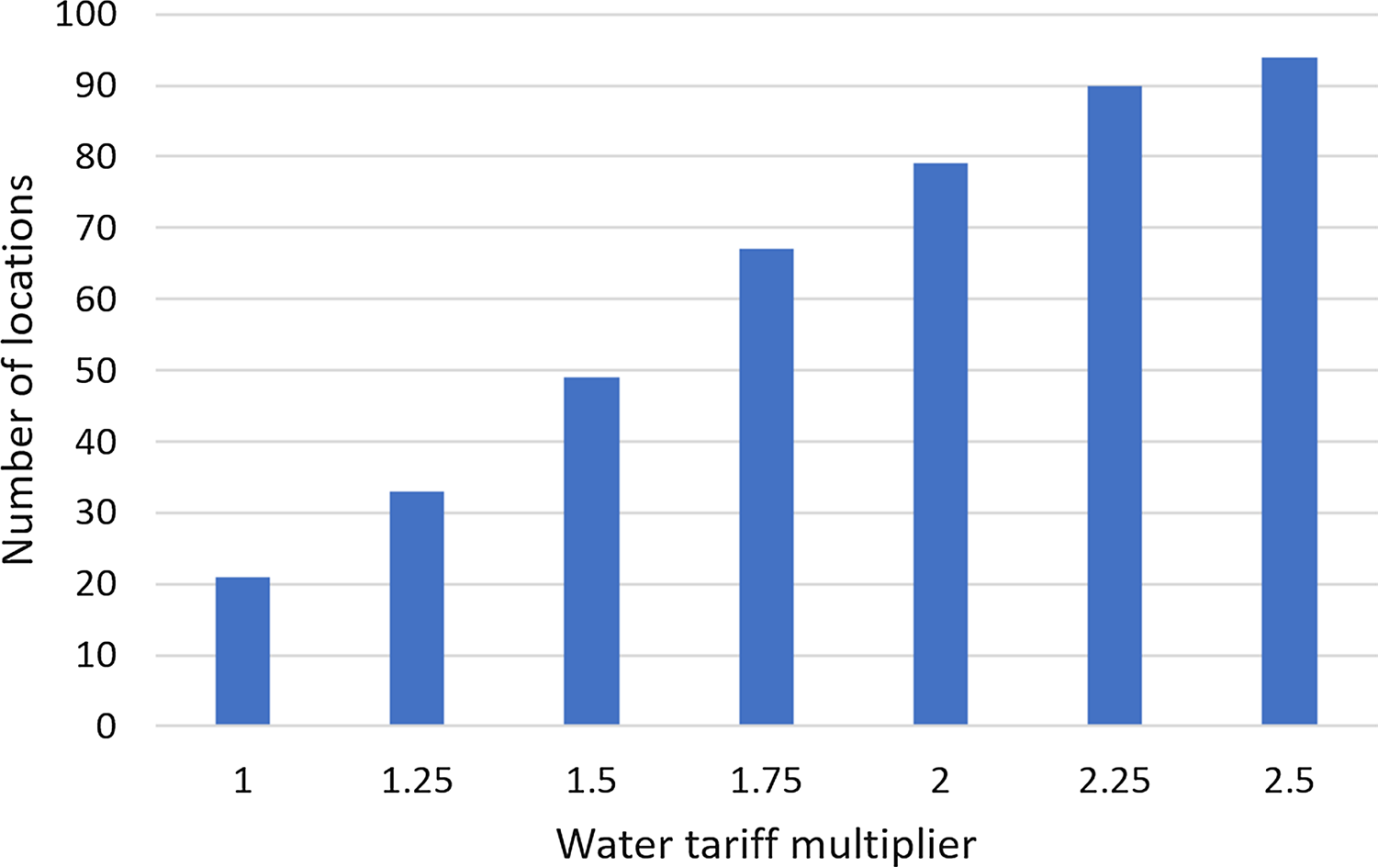
Effect of the water tariff multiplier on the number of locations with positive net present value for the 14-year period. Annual operating expenses: 1% of the capital expenditure. Catchment area: 150 m^2^; drainage coefficient: 0.85; number of occupants: 3.31; tank capacity: 5000 L; minimum volume allowed: 10% of the tank capacity; annual discount rate: 4%

Water tariffs are based on consumption tiers in many cities. While it is relatively simple to account for such tiers at a single location (e.g., [52]), price policies are city-dependent and this is information is unavailable in the public database [27] used as data source in this work, which only reports the base tariff. Here, we multiply this base tariff at each location by a water tariff multiplier. A multiplier equal to 2 means that the average water tariff is twice the base tariff listed in the National System for Sanitation Information [27]. We simulate the RWH system for different water tariffs, with the fixed values of the other variables shown in the caption of Fig. 13, which displays the effect of the water tariff multiplier on the number of locations with positive $NPV_{14}$. The number of locations with positive $NPV_{14}$ is rather sensitive to the average water tariff. For instance, this number of locations nearly quadruples when the water tariff multiplier changes from 1 to 2, changing from 14.2 to 53.4% of the 148 locations analyzed in this work. These locations belong to 11 states, mostly in the southern and southeastern regions, and to the Federal District (Brasília region). The importance of water tariffs on attractiveness identified here is aligned with previous observations of our own group regarding the city of Asunción, in Paraguay [52].

### Effect of catchment area

**Fig. 14:**
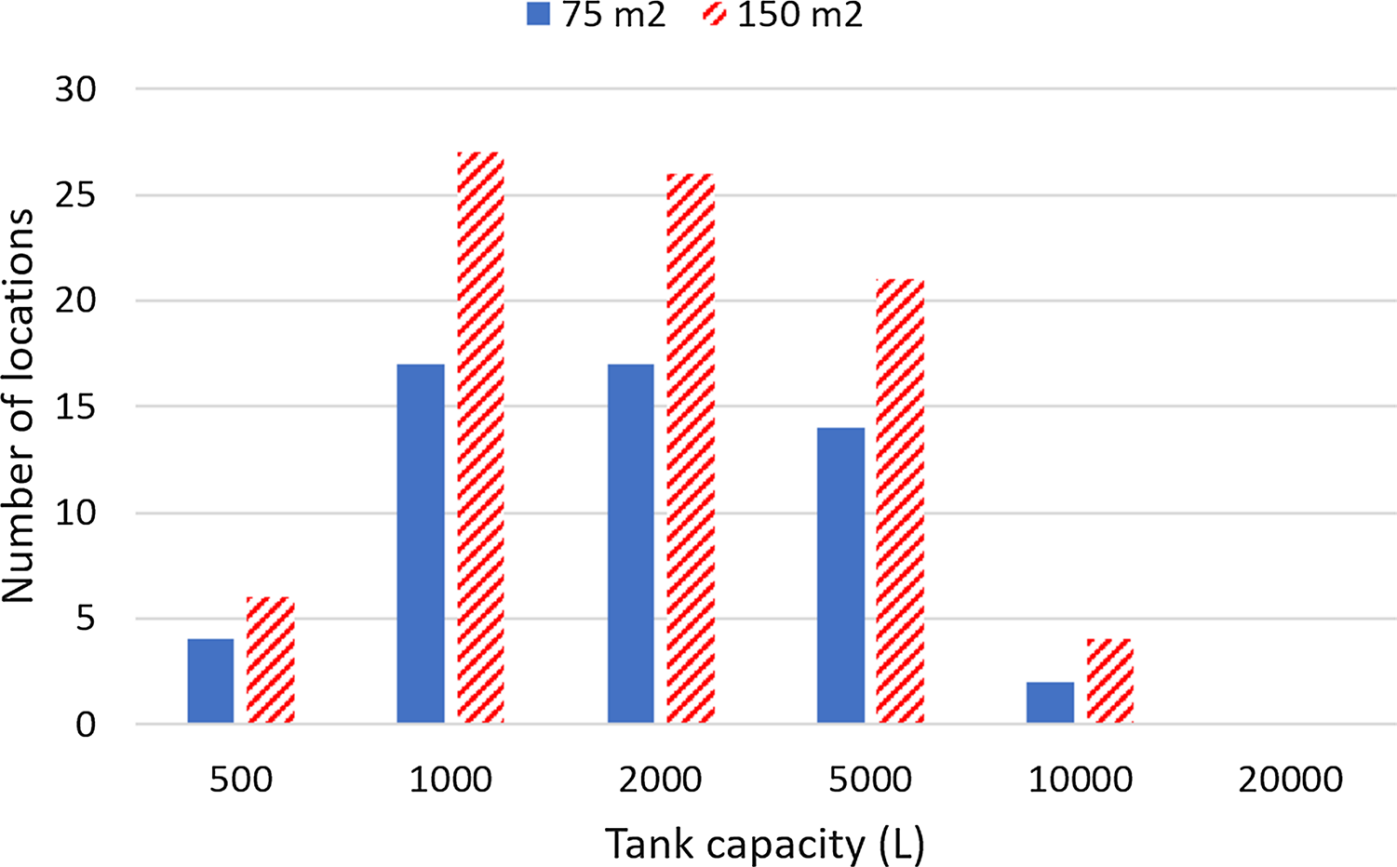
Effect of the tank capacity on the number of locations with positive net present value for the 14-year period. Annual operating expenses: 1% of the capital expenditure. Catchment area: 75 m^2^; drainage coefficient: 0.85; number of occupants: 3.31; minimum volume allowed: 10% of the tank capacity; annual discount rate: 4%

In 2021, the average area of the houses financed in Brazil was 66 m^2^ [47], to which we added 10% to account for the roof’s eaves. This results in a catchment area of 72.6 m^2^, which was rounded to 75 m^2^. In this way, this catchment area is half of the base case area of 150 m^2^. Figure 14 compares the number of locations with a positive $NPV_{14}$ for roofs with catchment areas of 75 m^2^ and 150 m^2^, for various water tank capacities. The additional specifications to run these cases can be found in the caption of Fig. 14.

For the 20,000 L tank, none of the two catchment areas considered gives a location with positive $NPV_{14}$. For the other tank capacities, the catchment area of 150 m^2^ leads to more locations with positive $NPV_{14}$ than the 75 m^2^ catchment area. As the 75 m^2^ catchment area is representative of the average house financed in Brazil in 2021 [47], this result suggests the economic attractiveness of domestic RWH systems will depend on favorable conditions related to occupancy, equipment cost, and water tariffs. Finally, it is also observed that cities of the state of Rio Grande do Sul are the most common among the locations with positive $NPV_{14}$ in Fig. 14.

## Conclusions

This work presented an economic assessment of rainwater harvesting for domestic applications in 148 Brazilian locations using historical rainfall data for the 14-year period 2008–2021, official statistical data for water consumption and tariff in each of these locations, and current equipment prices to assemble rainwater harvesting systems in Brazil. It is important to stress that the results reported in this paper only considered the economics of RWH systems from the perspective of a house owner. In households with 150 m^2^ and 3.31 occupants, even disregarding operating expenses and discount rates, only 58 locations, or about 39.2% of the total, had positive net present values for this 14-year period. When annual operating expenses equal to 1% of the capital expenditure were considered, the number of cities with positive net present values for this 14-year period dropped to 41, or about 27.7% of the total. When discount rates were also taken into account, the number of locations with positive net present values for this 14-year period dropped further: only 15 locations, about 10% of the total, when an annual discount rate of 8% was applied. In terms of geography, although many cities in the Brazilian northeast are in a semi-arid region, our base calculations suggest that the economic attractiveness of installing RWH systems there is low because of the little rainfall, small water consumption, and low water tariff. On the other hand, also according to our base case calculations, Brazil’s southernmost state of Rio Grande do Sul exhibits the most favorable conditions for the installation of RWH systems, as a result of the high water tariffs in its cities, compared to those in cities of other states.

This study also demonstrated that, compared to the base cases, higher occupancies, lower filtration equipment costs, larger water tariffs, and larger catchment areas affect the net present value for the 14-year period considered, generating scenarios that are favorable to the installation of domestic RWH systems in more locations in Brazil, particularly in cities of the southern and southeastern regions.

## Data Availability

The data files and the Python code used are available upon request.

## Appendix: Locations, rainfall, tariffs, and water consumption

See Table 2.

**Table 2:** Locations with state and INMET (Brazilian National Institute of Meteorology) code, latitude (°), longitude (°), average annual rainfall (mm), water tariff (US$/m^3^) [27], and consumption per capita (L/day/person) [27]

Location, state, INMET code	Latitude	Longitude	Rainfall	Tariff	Consumption
Águas Vermelhas, MG, A549	− 15.7464	− 41.4614	634.7	0.875	111.02
Aimorés, MG, A534	− 19.4966	− 41.0718	837.8	0.483	243.45
Alegre, ES, A617	− 20.7625	− 41.5317	1313.2	0.287	145.98
Alegrete, RS, A826	− 29.7838	− 55.7937	1504.8	1.732	131.98
Alfredo Chaves, ES, A615	− 20.6352	− 40.7500	1572.5	0.568	89.34
Almenara, MG, A508	− 16.1797	− 40.6954	778.3	0.981	129.49
Alto Paraíso de Goiás, GO, A024	− 14.1340	− 47.5140	1168.3	1.379	254.43
Apodi, RN, A340	− 5.6622	− 37.7989	662.2	0.929	104.87
Areia, PB, A310	− 6.9677	− 35.7026	1214.6	0.913	103.86
Arraial do Cabo, RJ, A606	− 22.9668	− 42.0278	884.8	2.149	151.31
Bagé, RS, A827	− 31.3333	− 54.1065	1441.7	0.958	131.9
Barbacena, MG, A502	− 21.2220	− 43.7724	1487.2	0.58	149.71
Barreiras, BA, A402	− 12.1477	− 44.9953	881.8	1.109	121.9
Bauru, SP, A705	− 22.3155	− 49.0708	1141.8	0.607	176.03
Belém, PA, A201	− 1.4563	− 48.5013	3333.8	0.818	117.64
Belo Horizonte, MG, A521	− 19.8527	− 43.9560	1675.1	1.193	156.22
Bento Gonçalves, RS, A840	− 29.1667	− 51.5170	1666.0	1.798	160.16
Bom Jardim da Serra, SC, A845	− 28.3403	− 49.6247	2854.1	1.332	106.73
Brasília, DF, A001	− 15.7975	− 47.8919	1471.5	1.117	142.03
Buritis, MG, A544	− 15.6288	− 46.4299	865.0	0.995	145.3
Cabrobó, PE, A329	− 8.5135	− 39.3096	409.1	0.776	89.71
Caçapava Do Sul, RS, A812	− 30.5147	− 53.4830	1662.7	1.877	127.49
Caldas, MG, A530	− 21.9235	− 46.3885	1368.9	1.106	164.79
Camaquã, RS, A838	− 30.8489	− 51.8070	1471.4	1.771	127.83
Campina Grande, PB, A313	− 7.2206	− 35.8888	584.5	0.861	117.49
Campina Verde, MG, A519	− 19.5340	− 49.4808	1362.8	1.059	150.3
Campo Grande, MS, A702	− 20.4649	− 54.6218	1433.1	1.327	168.41
Campos, RJ, A607	− 21.7615	− 41.3168	1001.2	1.158	110.62
Canguçu, RS, A811	− 31.3987	− 52.6777	1486.5	1.924	111.48
Capelinha, MG, A541	− 17.6934	− 42.5181	1045.5	0.956	124.57
Caracol, PI, A337	− 9.2837	− 43.3272	441.7	0.659	73.1
Caratinga, MG, A554	− 19.7889	− 42.1365	1107.0	1.071	136.77
Chapada Gaúcha, MG, A548	− 15.2985	− 45.6253	1051.0	0.96	122.19
Conceição das Alagoas, MG, A520	− 19.9242	− 48.3811	1261.4	0.45	163.9
Cristalina, GO, A036	− 16.7639	− 47.6085	1555.0	1.218	104.04
Cruz Alta, RS, A853	− 28.6440	− 53.6065	1773.1	1.707	141.71
Cuiabá, MT, A901	− 15.5954	− 56.0926	1262.7	0.871	177.91
Curitiba, PR, A807	− 25.4372	− 49.2700	1452.9	1.17	142.78
Curvelo, MG, A538	− 18.7703	− 44.4299	1129.9	1.107	133.63
Diamantina, MG, A537	− 18.2442	− 43.5980	1328.5	1.032	127.17
Dores do Indaiá, MG, A536	− 19.4614	− 45.5958	1273.0	1.061	153.18
Duque de Caxias, RJ, A603	− 22.5789	− 43.3150	1978.5	1.1	237.52
Erechim, RS, A828	− 27.6347	− 52.2747	1658.9	1.693	153.24
Espinosa, MG, A543	− 14.9209	− 42.8111	625.7	0.919	157.72
Feira de Santana, BA, A413	− 12.2536	− 38.9601	666.7	1.04	99.06
Florianópolis, SC, A806	− 27.5948	− 48.5569	1864.4	1.096	168.41
Formiga, MG, A524	− 20.4649	− 45.4263	1235.1	0.537	179.52
Frederico Westphalen, RS, A854	− 27.3587	− 53.3994	1906.2	2.071	161.04
Garanhuns, PE, A322	− 8.8909	− 36.4965	790.3	0.83	99.49
Goiânia, GO, A002	− 16.6869	− 49.2648	1425.7	1.202	149.21
Goiás, GO, A014	− 15.7050	− 49.3653	1475.2	1.23	151.57
Governador Valadares, MG, A532	− 18.8558	− 41.9572	947.6	0.652	149.59
Guanhães, MG, A533	− 18.7770	− 42.9380	1073.4	0.593	129.83
Gurupi, TO, A019	− 11.7244	− 49.0614	1363.1	1.131	136.17
Iguatu, CE, A319	− 6.3588	− 39.2981	844.1	0.661	115.84
Inácio Martins, PR, A823	− 25.5745	− 51.0752	1691.4	1.09	83.59
Indaial, SC, A817	− 26.8977	− 49.2363	1611.3	1.305	127.19
Itabaianinha, SE, A417	− 11.2750	− 37.7879	995.0	0.921	101.62
Itaberaba, BA, A408	− 12.5308	− 40.3084	437.9	1.115	96.28
Itaobim, MG, A550	− 16.5586	− 41.5026	696.2	1.014	135.37
Itapeva, SP, A714	− 23.9841	− 48.8812	1280.1	0.644	131.59
Itirucu, BA, A407	− 13.5349	− 40.1504	820.0	1.012	76.37
Ituiutaba, MG, A512	− 18.9746	− 49.4601	1303.1	0.541	193.85
Itumbiara, GO, A035	− 18.4167	− 49.2171	1115.7	1.152	158.09
Ivaí, PR, A818	− 25.0065	− 50.8549	1398.2	1.061	90.07
Jaguarão, RS, A836	− 32.5651	− 53.3779	1293.3	1.827	136.74
Jataí, GO, A016	− 17.8881	− 51.7264	1502.4	1.173	167.86
Joaçaba, SC, A841	− 27.1744	− 51.5054	1940.9	0.749	178.01
João Pinheiro, MG, A553	− 17.7406	− 46.1743	1129.0	1.086	145.01
Joaquim Távora, PR, A821	− 23.4988	− 49.9258	1237.6	1.117	128.04
Jose Bonifácio, SP, A735	− 21.0549	− 49.6785	1025.6	0.144	341.37
Juiz de Fora, MG, A518	− 21.7624	− 43.3435	1566.2	0.855	161.25
Lagoa Vermelha, RS, A844	− 28.2097	− 51.5299	1750.9	1.792	139.01
Linhares, ES, A614	− 19.3991	− 40.0654	1114.7	0.378	136.67
Lins, SP, A727	− 21.6733	− 49.7471	944.4	0.593	205.11
Luziânia, GO, A012	− 16.2506	− 47.9261	1303.5	1.218	92.14
Mal. Cândido Rondon, PR, A820	− 24.5562	− 54.0590	1500.9	0.721	197.47
Mantena, MG, A540	− 18.7834	− 40.9813	1056.5	0.318	161.44
Marambaia, RJ, A602	− 23.0689	− 43.9532	1092.1	1.265	166.27
Maria da Fé, MG, A531	− 22.3080	− 45.3735	1607.4	1.08	137.81
Maringá, PR, A835	− 23.4210	− 51.9331	1461.3	1.212	144.34
Montalvânia, MG, A526	− 14.4220	− 44.3674	749.9	0.997	139.42
Monte Alegre de Goiás, GO, A032	− 13.2573	− 46.8925	1074.9	1.356	101.23
Monteiro, PB, A334	− 7.8916	− 37.1239	578.1	0.753	77.57
Montes Claros, MG, A506	− 16.7292	− 43.8671	828.4	1.067	114.77
Morada Nova, CE, A332	− 5.1084	− 38.3720	570.1	0.733	122.36
Morrinhos, GO, A003	− 17.7356	− 49.1119	1261.3	1.189	158.97
Mossoró, RN, A318	− 5.1919	− 37.3424	596.4	0.966	105.02
Muriaé, MG, A517	− 21.1291	− 42.3702	1581.5	0.597	162.98
Natal, RN, A304	− 5.7842	− 35.2000	1551.5	0.896	118.4
Ouro Branco, MG, A513	− 20.5235	− 43.6914	1475.5	1.109	133.12
Palmas, TO, A009	− 10.2491	− 48.3243	1482.2	1.113	149.48
Parati, RJ, A619	− 23.2197	− 44.7167	1594.8	0.983	133.07
Passo Fundo, RS, A839	− 28.2623	− 52.4103	1747.3	1.74	149.77
Passos, MG, A516	− 20.7221	− 46.6133	1170.1	0.508	198.29
Patos, PB, A321	− 7.0263	− 37.2770	679.7	0.886	109.23
Patrocínio, MG, A523	− 18.9380	− 46.9943	1350.5	0.646	123.72
Paulistana, PI, A330	− 8.1360	− 41.1417	518.0	0.762	93.83
Pedro Afonso, TO, A020	− 8.9755	− 48.1736	1638.8	0.223	387.43
Petrolina, PE, A307	− 9.3995	− 40.5024	388.5	0.871	108.8
Piracicaba, SP, A726	− 22.7343	− 47.6481	1216.7	0.741	199.55
Pirapora, MG, A545	− 17.3489	− 44.9468	979.0	0.652	180.85
Pires do Rio, GO, A033	− 17.2997	− 48.2822	1237.0	1.232	154.74
Ponta Porã, MS, A703	− 22.5373	− 55.7282	1665.0	1.086	139.24
Porto Alegre, RS, A801	− 30.0368	− 51.2090	1529.1	0.972	215.29
Posse, GO, A017	− 14.0920	− 46.3649	1061.5	1.206	122.41
Presidente Prudente, SP, A707	− 22.1208	− 51.3873	1208.0	0.685	187.22
Quaraí, RS, A831	− 30.3799	− 56.4522	1361.1	1.74	131.21
Resende, RJ, A609	− 22.4681	− 44.4479	1552.9	0.654	164.37
Rio de Janeiro, RJ, A652	− 22.9862	− 43.1865	1148.0	1.265	166.27
Rio de Janeiro, RJ, A621	− 22.8648	− 43.3998	1117.3	1.265	166.27
Rio Grande, RS, A802	− 32.0408	− 52.1017	1141.6	1.808	129.17
Rio Pardo de Minas, MG, A551	− 15.6151	− 42.5433	771.2	1.028	104.03
Rio Pardo, RS, A813	− 29.9844	− 52.3760	1497.1	1.779	145.85
Rio Verde, GO, A025	− 17.7896	− 50.9205	1344.7	1.175	130.76
Sacramento, MG, A525	− 19.8661	− 47.4410	1495.3	0.479	156.8
Salinas, MG, A552	− 16.1684	− 42.2923	716.3	0.877	130.41
Santa Maria, RS, A803	− 29.6895	− 53.7923	1804.6	1.763	133.81
Santa Rosa, RS, A810	− 27.8709	− 54.4833	1673.6	1.736	167.74
Santa Teresa, ES, A613	− 19.9320	− 40.5999	1607.6	0.793	157.22
Santo Augusto, RS, A805	− 27.8509	− 53.7763	1812.1	1.72	152.84
São Carlos, SP, A711	− 22.0123	− 47.8908	1451.7	0.626	244.96
São Gabriel, RS, A832	− 30.3344	− 54.3218	1525.1	1.358	139.72
São João del Rei, MG, A514	− 21.1362	− 44.2576	1472.3	0.155	431.49
Sao José dos Ausentes, RS, A829	− 28.7488	− 50.0653	1592.7	1.808	118.65
São Luís Do Paraitinga, SP, A740	− 23.2219	− 45.3106	1363.8	0.712	154.93
São Luiz Gonzaga, RS, A852	− 28.4081	− 54.9615	1827.2	1.72	141.82
São Mateus, ES, A616	− 18.7166	− 39.8559	1157.3	0.617	95.92
São Paulo-Mirante, SP, A701	− 23.4683	− 46.7217	1593.9	0.762	164.7
São Romão, MG, A547	− 16.3646	− 45.0753	901.1	0.97	119.97
Seropédica, RJ, A601	− 22.7472	− 43.6996	1190.5	1.524	313.16
Serra dos Aimorés, MG, A522	− 17.7886	− 40.2446	891.5	1.005	116
Sorocaba, SP, A713	− 23.4709	− 47.4851	1163.5	0.576	188.23
Tauá, CE, A324	− 6.0033	− 40.2946	501.2	0.78	119.36
Teófilo Otoni, MG, A527	− 17.8608	− 41.5090	1049.7	1.102	133.92
Teresópolis, RJ, A618	− 22.4162	− 42.9720	2867.1	1.009	142.96
Timóteo, MG, A511	− 19.5824	− 42.6450	1356.3	1.115	122.38
Torres, RS, A808	− 29.3384	− 49.7282	1578.9	1.982	199.6
Três Marias, MG, A528	− 18.2053	− 45.2275	1207.1	1.09	140.69
Uberlândia, MG, A507	− 18.9128	− 48.2755	1431.1	0.378	241.34
Unaí, MG, A542	− 16.3647	− 46.8920	1180.0	0.45	196.17
Uruguaiana, RS, A809	− 29.7584	− 57.0863	1403.9	1.173	118.95
Valença, RJ, A611	− 22.2459	− 43.7069	1184.2	0.756	264.88
Valparaíso, SP, A734	− 21.2246	− 50.8657	1234.8	0.491	175.31
Varginha, MG, A515	− 21.5560	− 45.4364	1171.5	1.166	154.48
Viçosa, MG, A510	− 20.7561	− 42.8800	1356.2	0.613	149.55
Vitória, ES, A612	− 20.3197	− 40.3385	1503.5	0.929	201.24
Votuporanga, SP, A729	− 20.4183	− 49.9763	1368.6	0.498	200.24
